# Dental College Affiliates with Cincinnati University

**Published:** 1923-07

**Authors:** 


### DENTAL COLLEGE AFFILIATES WITH CINCINNATI UNIVERSITY.

The Ohio College of Dental Surgery, Seventh and Mound streets, became affiliated with the University of Cincinnati by unanimous action of the Board of Directors of the University, meeting in special session yesterday afternoon. This action was taken by the Board of Directors after a survey extending over a year. Dr. H. T. Smith, Dean of the Ohio College of Dental Surgery, co-operated with members of the board and Dr. Frederick C. Hicks, President, in investigating the feasibility of this affiliation.

The following report of the committee of the Board of Directors was adopted by unanimous vote:

"It is agreed that it is desirable that an affiliation be arranged between the University of Cincinnati and the Ohio College of Dental Surgery, to become effective for the ensuing academic year, on the following general conditions:

"1. The personnel of the faculty, the educational standards and the budget of the Ohio College of Dental Surgery shall be approved jointly by the college and university.

"2. All extra expenses involved in perfecting the affiliation shall be paid, until otherwise agreed upon, from funds provided by the college.

"3. Details pertaining to interchange of facilities between the college and the university shall be determined by agreement as the practical aspects of the affiliation are developed.

"4. Any surplus of income over expenditures shall be used solely for the promotion of the work of the college.

"5. The affiliation shall be discontinued by the university if, after a fair trial, adequate resources on which to make the college an integral part of the university and to develop the college into a Class A dental school, should not be available.

"We recommend that such an affiliation be approved and that the Committee on Law be instructed to draw up a contract therefor."

The motion for the affiliation was made by Arthur R. Morgan and seconded by Alfred Mack.

The Committee on Law, consisting of former Judge Rufus B. Smith, Chairman of the Board of Directors, Frank Dinsmore, Alfred Mack and Sandford Brown, were authorized by the board to draw up a contract for the affiliation of the two schools.

It was pointed out at the meeting that members of the dental profession in Cincinnati were heartily in favor of the step that had been taken by the University Board of Directors and Dean Smith of the Dental College.

The affiliation will become effective September 1st at the beginning of the next school year, in time for the opening of both the dental college and the university.

The Ohio College of Dental Surgery is the oldest dental college in the world. It was chartered by an act of the Legislature in 1845 and has been in continuous operation since then. Prof. Jesse W. Cook was elected the first Dean of the Ohio College of Dental Surgery.—*Enquirer*.

